# A Bibliometric Analysis of Published Literature in Postoperative Pain in Elderly Patients in Low- and Middle-Income Countries

**DOI:** 10.3390/jcm10112334

**Published:** 2021-05-27

**Authors:** João Batista Santos Garcia, Érica Brandão de Moraes, José Osvaldo Barbosa Neto

**Affiliations:** 1 Department of Anesthesiology, Pain and Palliative Care, Universidade Federal do Maranhão, São Luís 65080-805, Brazil; 2 School of Nursing, Universidade Federal Fluminense, Niteroi 24020-091, Brazil; ericabrandao@id.uff.br; 3 Faculty of Medicine, Universidade CEUMA, São Luís 65075-120, Brazil; jose.barbosa@ceuma.br

**Keywords:** elderly, postoperative pain, developing countries, low-income countries, middle-income countries, bibliometric analysis, bibliometric review, Scopus, Web of Science

## Abstract

Postoperative pain (POP) remains a major challenge for surgeons and anesthesiologists worldwide, especially in low- and middle-income countries. Elderly patients are at higher risk for undertreatment of pain. Despite that, there is a paucity of papers addressing POP among this population in developing countries. This study aimed to provide a bibliometric analysis of the literature concerning postoperative pain in elderly patients from low- and middle-income countries. It was performed an extensive search of papers on this subject through the Web of Science and Scopus database using a series of uniterms and, including publications from 2001 to 2021. Publication quality was assessed by using total citation frequency, average citations per item and other citation indexes. Citation indexes were low, with the highest reaching 15 citations. In conclusion, few studies of postoperative pain in the elderly in countries with medium and low income, indicating a need that has not yet been met for this population and in these areas of the world. The published studies were not specifically aimed at the elderly, had limited impact, low international visibility. They were not epidemiological studies and are not robust, weakening knowledge and decision-making towards policies directed at this vulnerable population.

## 1. Introduction

Postoperative pain (POP) remains a major challenge for surgeons and anesthesiologists worldwide, especially in low- and middle-income countries, due to important limitations, such as technological and human resources and availability of analgesics [1,2,3,4]. POP incidence available in the literature usually reflects data from specific surgeries or reflects regional realities and, therefore, presents great variability, showing averages ranging from 14–62% [1,2,5,6]. When looking for data from low- and middle-income countries, these numbers are scarce and frequently come from single-center or small samples studies [1,2,4,7].

Due to their specific characteristics, some populations have a higher risk of having their pain poorly evaluated, and therefore, undertreated. The elderly population accumulates different aspects that lead to a greater risk of POP: they may have chronic pain due to other clinical situations, which can confuse their evaluation in the postoperative period [8], there may be a limitation in using analgesics due to fear of adverse effects, and often, the elderly refrain from reporting their pain to avoid disturbing the care team [9].

These factors may explain why in a European retrospective multicenter study that evaluated risk factors for postoperative pain, younger patients appeared to be at higher risk than the elderly [10]. Contradictorily, in a study conducted in the United States that compared postoperative pain at different ages, elderly patients had more pain and received more analgesics [11]. The main difference between these studies is that in the latter, patients had their pain evaluated through individual interviews rather than from a database. By doing that, the confounding factors described earlier could be avoided.

Bibliometric studies (use of quantitative methods in the search for an objective assessment of the scientific production of knowledge) and revisions of the literature (in its different types) are essentially important for evaluating science and information flows since they are types of studies that seek to determine the state of art or knowledge [12]. In this context, bibliometric indicators have become essential because it has been clear the importance of presenting a distribution that informs about the number of authors, works, countries, scientific links, journals, and impact factors of publications that exist in each category of productivity. Therefore, these indicators can be at the heart of the debates, both in terms of the relationship between the advancement of science and technology and in economic and social progress [12].

Considering the advances in the knowledge of postoperative pain in recent decades, the importance of mapping and discussing academic production in this area of knowledge is emphasized, especially in vulnerable populations, such as the elderly and in regions of the world with the greatest need for resources. The monitoring of this production needs to be carried out in parallel, since, by identifying and explaining the paths taken, it may be possible to identify the process of building knowledge on this very important topic, observing duplications, contradictions and, especially, gaps, that is, aspects not yet explored.

Considering the vulnerability of the elderly population and the paucity studies evaluating postoperative pain directed specifically to those patients, a bibliometric review was carried out to assess the quantity and quality of research on postoperative pain in older patients from low- and middle-income countries.

## 2. Materials and Methods

### 2.1. Data Sources

Bibliometric review is a set of methods to quantitatively analyze the bibliographic data of published scientific literature to provide an overview of the body of knowledge for a given field of inquiry.

All data were acquired on 2 February 2021. Elderly postoperative pain in countries with limited resources-related articles published in peer-reviewed journals between 2001 and 2021 was retrieved from Scopus and the Web of Science (WOS) as the main database to conduct our search.

Web of Science (owned by Clarivate, formerly Thomson Reuters) and Scopus (owned by Elsevier) are the best-known sources of curated citation data for subscribing institutions. The combination of Web of Science e Scopus database covers more scientific disciplines, a broader range of dates of publications, a large spread of countries and provides a detailed citation analysis. Moreover, these bases are the ones used in the bibliometric analysis [13,14].

### 2.2. Search Strategy

The search strategy was as comprehensive as possible to identify all publications. A keyword mapping was performed to structure the search: (“pain, postoperative” OR “postsurgical pain” OR “pain, postsurgical” OR “post surgical pain” OR “pain, postoperative” OR “pain, post operative” OR “postsurgical pain” OR “pain, postsurgical” OR “postoperative pain” OR “post operative pain” OR “postoperative pains” OR “postoperative pain” OR “postoperative pain, acute” OR “pain, acute postoperative” OR “acute postoperative pain” OR “acute postoperative pain” OR “acute post operative pain” OR “postoperative pain, acute” OR “pain, acute postoperative” OR “post operative pain, acute”) AND (aged OR elderly) AND (“developing countries” OR “countries, developing” OR “country, developing” OR “developing country” OR “least developed countries” OR “countries, least developed” OR “country, least developed” OR “developed countries, least” OR “developed country, least” OR “least developed country” OR “less-developed countries” OR “countries, less-developed” OR “country, less-developed” OR “less developed countries” OR “less-developed country” OR “under-developed nations” OR “nation, under-developed” OR “nations, under-developed” OR “under developed nations” OR “under-developed nation” OR “third-world countries” OR “countries, third-world” OR “country, third-world” OR “third world countries” OR “third-world country” OR “third-world nations” OR “nation, third-world” OR “nations, third-world” OR “third world nations” OR “third-world nation” OR “under-developed countries” OR “countries, under-developed” OR “country, under-developed” OR “under developed countries” OR “under-developed country” OR “developing nations” OR “developing nation” OR “nations, developing” OR “less-developed nations” OR “less developed nations” OR “less-developed nation” OR “nation, less-developed” OR “nations, less-developed”). In this search, there was no limitation regarding the specific type of study design, nor the type of publication.

Publication quality was assessed by using total citation frequency, average citations per item and H-index, CiteScore, SCImago journal rank (SjR) and source-normalized impact per paper (SNIP). Literature quantity and publication trend were analyzed by total publications, research types, research organization, author’s contribution, journal, and funding support. Related data, such as the number of publications, citations, H-index, journal, reference, and keyword, were extracted and recorded as bibliometric indicators.

### 2.3. Bibliometric Analysis

In this study, a quantitative descriptive analysis based on bibliometric analysis was performed, and the data produced were presented through graphs and tables containing results in absolute numbers and percentages.

Bibliometric mapping and clustering analysis were performed using the VOS visualization software, a software tool for building and visualizing bibliometric networks. Bibliometric mapping allows visualization of academic output in terms of publications and citation information for a particular field. Cluster analysis uses a series of algorithms to detect the natural division of research group networks (clusters) based on similarity and allows visualization of co-authorship networks between researchers, institutions, and countries.

## 3. Results

### 3.1. Articles from Low- and Middle-Income Countries: Number and Type of Publications, Authors, Affiliations, Distribution of Countries and Grants Awarded

Eighty studies resulted from searching the Scopus and Web of Science databases. Twenty of them were duplicated and were deleted. After further evaluation of the 60 remaining papers, only 15 were included in the bibliometric data analysis [15,16,17,18,19,20,21,22,23,24,25,26,27,28,29]. The main reasons for exclusion were: 28 articles—age group with adults; 4 articles—the age group with children; 6 articles—had no postoperative pain; 4 articles—evaluated chronic pain; 3 articles—addressed surgical technique and costs. The studies, distributed by continent, were 6 from Africa, 2 from South America and 7 from Asia. Table 1 shows the characterization of the studies included in the review. Of the 80 studies recovered, only 15 papers met the criteria to be included. Most articles are from African and Asian nations, and over one-third of them had international collaboration.

**Figure 1:**
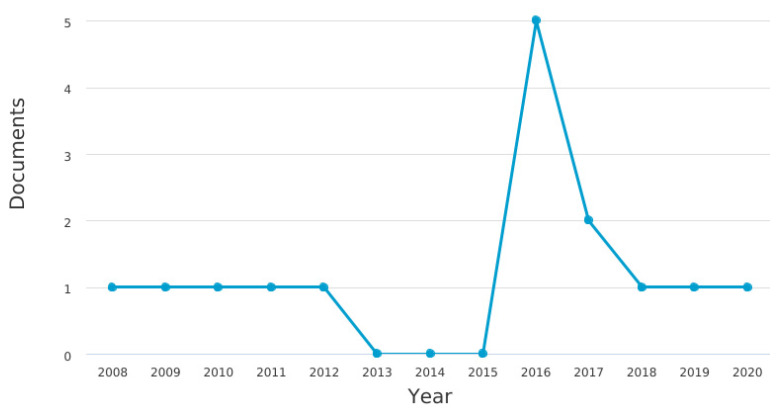
Number of publications on postoperative pain over the years.

There was a low number of publications in most years, with one publication/year, and reaching the peak of publications in 2016 (5 publications/year). Figure 1 shows the number of publications on the topic over the years.

**Figure 2:**
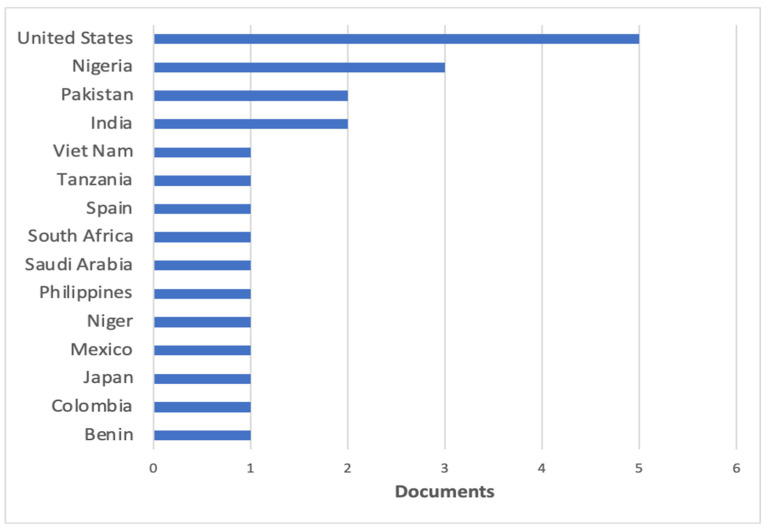
Publications by country or territory.

Twenty-three countries were cited in the authors’ affiliations section of the 15 publications included in this review. Many of the selected articles had contributions from authors from developed countries. Among them, the United States leads in a number of participations. The countries that contributed with the largest number of studies were Nigeria (3 publications), India (2 publications) and Pakistan (2 publications) (Figure 2).

### 3.2. Citations and Index Analysis

One of the most cited publications, with a robust sample, is a study that analyzed the acute postoperative pain in 1231 patients at a developing country referral hospital in South Africa. Table 2 shows the number of citations per year in each of the publications and the sum of citations of the top 5 publications over the years.

**Figure 3:**
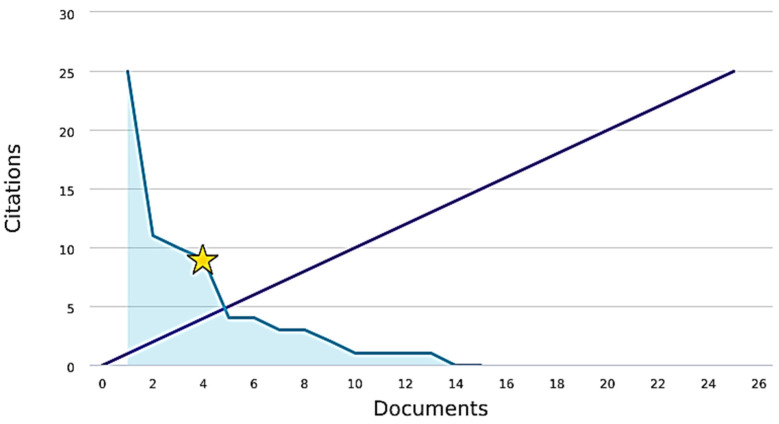
H-index of publications.

The *h*-index is an index that attempts to measure both the productivity and impact of the published work of a scientist or scholar. It is based on the highest number of papers included that have had at least the same number of citations. The graph shows a 45 degree line, which models a 1:1 relationship between publishing articles and being cited. Figure 3 shows that of the 15 documents considered for the h-index, 4 were cited at least 4 times.

**Figure 4:**
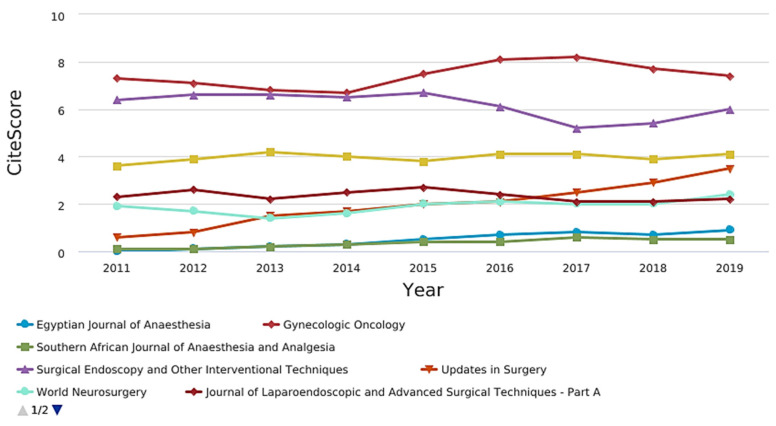
CiteScore of pain, anesthesia, and surgery journals.

**Figure 5:**
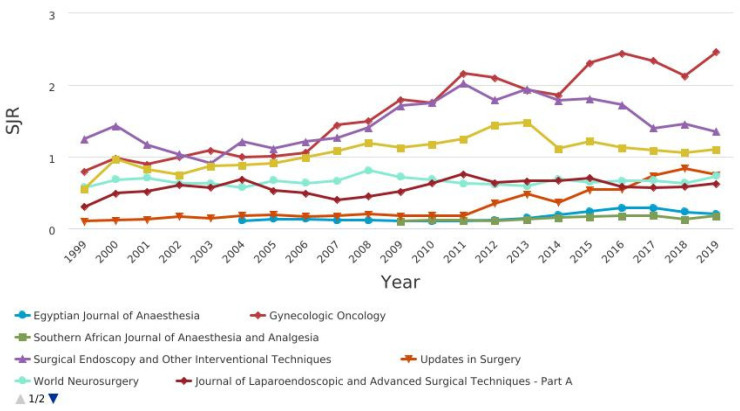
SCImago journal rank (SjR) of pain, anesthesia, and surgery journals.

**Figure 6:**
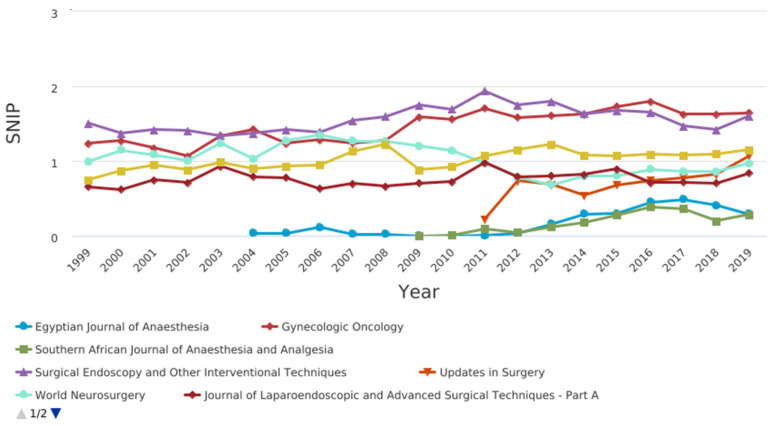
Source-normalized impact per paper (SNIP) of pain, anesthesia, and surgery journals.

**Figure 7:**
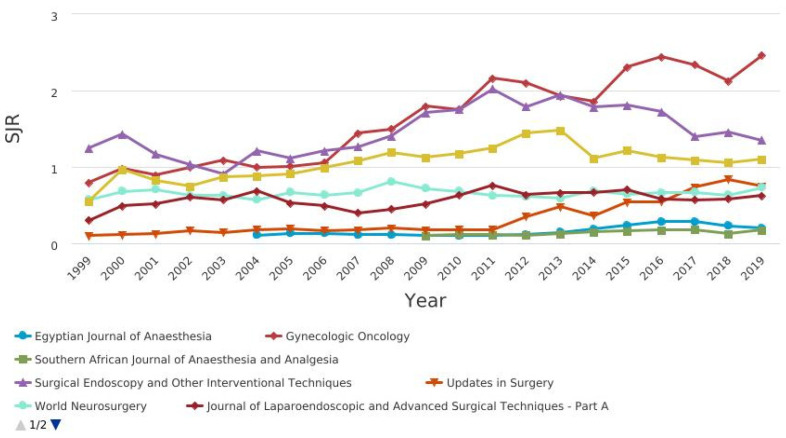
SCImago journal rank (SjR) of pain, anesthesia, and surgery journals.

**Figure 8:**
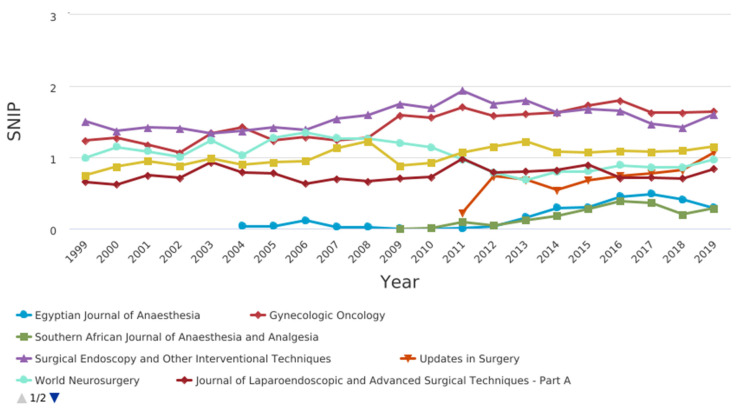
Source-normalized impact per paper SNIP) of pain, anesthesia, and surgery journals.

Figure 4 shows the CiteScore graph.Among the journals, those related to pain, anesthesia and surgery were compared in terms of citation and impact factor (Figure 5, Figure 6, Figure 7 and Figure 8). 

CiteScore** is** the number of citations received by a journal in one year to documents published in the three previous years, divided by the number of documents indexed in Scopus published in those same three years. The journals with the best scores over the years are “Gynecologic Oncology” and “Surgical Endoscopy and Other Interventional Techniques”. Figure 7 shows the SCImago journal rank (SjR) graph. It expresses the average number of weighted citations received in the selected year by the documents published in the selected journal in the three previous years. The journals with the best scores over the years are “Gynecologic Oncology” and “Surgical Endoscopy and Other Interventional Techniques”. Figure 8 shows the Source-normalized impact per paper SNIP) graph. It expresses a corrective metric to account for differences in citation potential in different fields. The journals with the best scores over the years are “Gynecologic Oncology” and “Surgical Endoscopy and Other Interventional Techniques”.

### 3.3. Analysis of Word Density

**Figure 9:**
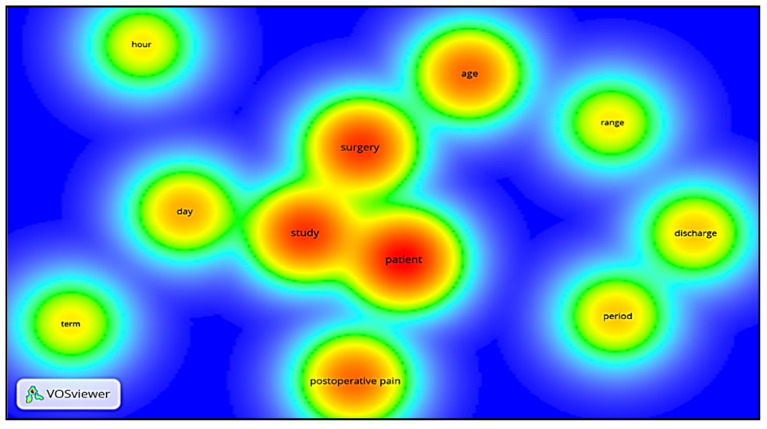
Word density visualization.

Figure 9 shows the word density visualization performed in the VOSviewer software by analyzing the words contained in the title and summary of publications. The system mapped 612 terms. The minimum criterion for selection was the occurrence of the term at least 4 times in the articles, with 60% relevance. Eleven words were selected, the words with a reddish center have a higher density.

### 3.4. Analysis of Clusters of Authors

**Figure 10:**
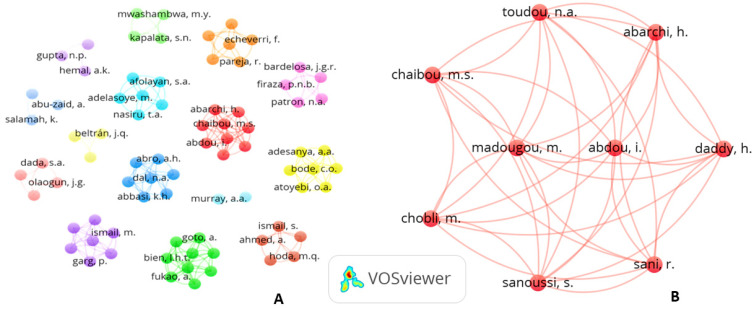
Clusters of authors. (**A**) Connection strength between authors (**B**) Map of the network of authors.

After analyzing the authors and co-authors, 80 names and 15 potential clusters of authors were identified (A). When analyzing the connection strength between the 80 names, it was observed that the largest set of clusters connected consists of 9 names (B) Figure 10.

## 4. Discussion

In the present study, we completed a bibliometric study of the scientific publications in postoperative pain research in elderly patients from low- and middle-income countries in the last twenty years, which helped us to comprehend the trend in this type of pain research in these areas of the world.

This review made evident the scarce number of publications in high-impact peer-reviewed journals, which are present in indexed databases. Differences in the number of studies published by authors from different regions of the world were also clear, with a predominant gap in publications from Latin American countries, especially Central America and the Caribbean. A point that draws much attention among the articles analyzed is the topic of laparoscopic operations, which was quite prominent. The emphasis given to this theme suggests an ongoing pursuit for improving postoperative pain control for laparoscopic surgery. In this review, the same interest was not observed towards postoperative pain in the elderly population. In this specific aspect, it is worth noting that all articles included elderly patients but were not directly addressed to them. This demonstrates that there are still limitations in preventing the suffering of this vulnerable group during the postoperative period, especially in countries where there is already a restriction of resources for the adequate treatment of pain [30].

There was an isolated peak of publications in 2016, which reached the shy number of five articles, returning later to a recurring basis of one publication per year. When added to those previously mentioned, these data reinforce the precariousness of information regarding postoperative pain in the elderly in these countries and strongly suggest the need for change in this very dramatic curve of publications.

To carry out studies in regions with few resources, international collaboration is important, especially with developed countries, which will allow technical cooperation in the most diverse areas, such as the elaboration of large-scale research projects, in the statistical analysis, in the contribution of financial resources, among others [31,32]. In this bibliometric analysis, the contribution of researchers from developed countries, such as the United States (5 studies), Spain (1 article) and Japan (1 article), was observed in part of the selected articles. The studies with international cooperation were published in journals of greater impact, such as Surgical Endoscopy, and therefore, obtained a higher number of citations. Of the fifteen articles in the review, only two declared funding, one private and the other governmental, reflecting a lack of support for this type of research.

None of the articles that ranked among the five most cited in the present review received over 15 citations since they were published, a very small number, and that reflects that these publications did not obtain significant international visibility. Compared to the five most cited articles in the field of anesthesiology, the total number of citations of these ranged from 466 to 699 [33]. None had postoperative pain as their main theme, and all were developed in developed countries. The article focusing on postsurgical pain, which had the highest number of citations, reached only 12th place and was also carried out in a developed country and did not specifically addressed the elderly population [33]. The data obtained in these two bibliometric reviews expose the problem that postoperative pain in elderly patients in developing countries is far from being discussed by the global scientific community based on robust epidemiological data.

Another noticeable result of this bibliometric review is the low impact of the publications, observed by the H-index. Still, when other citations and impact indexes were evaluated (CiteScore, SCImago journal rank and source-normalized impact per paper), directed to specific areas of pain, anesthesia, and surgery, it was observed that the surgery stood out over the other two areas. The most important publications were in surgery-oriented journals, and of the top five articles, only one was published in an anesthesia journal. Why does this gap exist in pain and anesthesia in these low- and middle-income countries? Several possible reasons and barriers could be suggested: a small number of indexed journals of anesthesiology and pain in these regions might be one. Among the journals focused on anesthesia that appear among the most cited, those that originate in developing countries did not reach citation averages greater than 3, while those with the highest averages exceed 20 [33]. Another possibility is the lack of quality in publications that allow them to achieve merit for publication in specific and high impact journals; the lack of encouragement for researchers from these countries to submit articles in high impact journals. In addition, the need for greater financial and technical support to help improve the quality of studies to be published; and the focus of scientific journals on more specific, innovative, and contemporary themes that occur in developed countries, to the detriment of the reality of less favored countries. This last barrier reinforces the concept of the 90/10 gap, which says that 90% of spending on medical research benefits only 10% of the world population. In general, these resources are used in developing technologies and treatments of high-cost, with low penetration in countries with less financial resources. Therefore, it is not always possible to directly implement the results of studies carried out in developed countries for countries with low- and medium-income [34]. Developing countries, according to a bibliometric study, had a small contribution to publications on anesthesia topics. Data referring specifically to postoperative pain or in special populations are not available to date [33].

In the analysis of the density of the words, a flaw/gap with the word “developing countries/with limited resources” is perceived, as well as postoperative pain, which despite being terms that were used in the search and that guided the studies regarding their location, were little used in the title and summary. Mapping more relevant words in the title and abstract can support new bibliometric analyses on the subject. The use of terms that reflect the research area in these parts of the articles is of paramount importance since it is one of the main search strategies of the databases [35].

In the authors’ network, greater collaboration was observed between researchers from the same institution than between authors from different countries in a specific region, that is, Asians, Latinos and Africans, they did not cooperate with each other in this bibliometric analysis. This might suggest regional isolation of the researchers, who did not obtain interchange and interinstitutional support in these studies in these countries. Exceptions were noted, such as cooperation between authors from Japan with Vietnam and the USA with Mexico and countries in Africa and the Middle East.

This study is the first bibliometric analysis to evaluate trend and development potential in the field of elderly postoperative pain research in the recent two decades in countries with limited resources. This research includes analysis of the number of publications, citations, H-index and others, journals and collaboration analysis between countries/institutions, analysis of authors and the density of words. This study has some limitations. Web of Science and Scopus are the only electronic database searched, and non-English publications were excluded. Perhaps articles with this theme and in these countries may have been published in non-indexed journals on the analyzed bases or on regional bases, such as LILACS, which concentrates on publications from Latin America.

In conclusion, there are few studies of postoperative pain in the elderly in countries with medium and low income, constituting a need that has not yet been met for this population and in these areas of the world. The published studies were not specifically aimed at the elderly, had limited impact, low international visibility and were not epidemiological studies. They are not robust, weakening knowledge and decision-making towards policies directed at this vulnerable population. New studies must be conducted, and stimuli to publications of different natures are needed. Cooperation between institutions and between countries is desirable to construct a solid basis for assessing postoperative pain in the elderly in low- and middle-income countries.

## Figures and Tables

**Table 1:** Characterization of the studies included in the review.

Author	Title	Type	First Author Affiliation	Grants
Balogun O.S.; et al. [25]	Development and practice of laparoscopic surgery in a Nigerian tertiary hospital	Article	Department of Surgery, College of Medicine University of Lagos/Lagos University Teaching Hospital, Idi-ArabaLagos, Nigeria	No
Flores R.E.; et al. [23]	An Affordable and Feasible Technique for Minimally Invasive Tubular Lumbar Discectomy	Article	Department of Neurosurgery, Hospital General de Zona No.7 IMSS, Monclova, Mexico	No
Mwashambwa M.Y.; et al. [18]	Post-operative pain prevalence, predictors, management practices and satisfaction among operated cases at a regional referral hospital in Dar es Salaam, Tanzania	Article	School of Medicine and Dentistry, College of Health Sciences, The University of Dodoma, P.O Box 395, Dodoma, Tanzania	No
Salamah K.; et al. [21]	Single-incision laparoscopic surgery in gynecologic surgery: A single-institutional experience from Saudi Arabia	Article	Department of Obstetrics and Gynecology, Women’s Specialized Hospital, King Fahad Medical City, Riyadh, Saudi Arabia	No
Abro A.H.; et al. [24]	Demographical evaluation of laparoscopic versus open appendectomy at tertiary care teaching hospital	Article	Department of Surgery, Liaquat University of Medical & Health Sciences (LUMHS), Jamshoro, Sindh, Pakistan	No
Ismail S.; et al. [19]	Prospective survey to study factors which could influence same-day discharge after elective laparoscopic cholecystectomy in a tertiary care hospital of a developing country	Article	Department of Anaesthesia, Aga Khan University Hospital, Stadium Road, PO Box 3500, Karachi, 74800, Pakistan	No
Rendón G.J.; et al. [29]	Outpatient laparoscopic nerve-sparing radical hysterectomy: A feasibility study and analysis of perioperative outcomes	Article	Department of Gynecologic Oncology, Instituto de Cancerología—Las Américas, Medellín, Colombia	No
Olaogun J.G.; et al. [15]	The feasibility and acceptability of day case surgery in secondary health facility in Nigeria	Article	Department of Surgery, Ekiti State University, Ado-Ekiti, Nigeria	No
Firaza P.N.B.; et al. [26]	Hybrid Natural Orifice Translumenal Endoscopic Surgery Transvaginal Nephrectomy in a Low-Resource Setting	Article	Department of Urology, Jose R. Reyes Memorial Medical Center, San Lazaro Compound, Rizal Avenue, Sta. Cruz, Manila, 1003, Philippines	No
Murray A.A.; et al. [22]	Acute postoperative pain in 1231 patients at a developing country referral hospital: Incidence and risk factors	Article	Department of Anaesthesiology and Critical Care, Stellenbosch University, Tygerberg, South Africa	Yes
Chaibou M.S.; et al. [27]	Management of postoperative pain: Experience of the Niamey National Hospital, Niger	Article	Department of Anesthesiology and Intensive Care, The Niamey National Hospital, Niamey, Niger	No
Garg et al. [28]	Mesh fixation compared to nonfixation in total extraperitoneal inguinal hernia repair: A randomized controlled trial in a rural center in India	Article	Department of General Surgery, MM Institute of Medical Sciences and Research, Mullana, Haryana, India	No
Soejima K.; et al. [17]	Perception of anesthesia safety and postoperative symptoms of surgery patients in Ho Chi Minh City, Vietnam: A pioneering trial of postoperative care assessment in a developing nation	Article	Department of Public Health, Faculty of Medicine, Yamagata University, Yamagata, Japan	No
Kumar A.; et al. [16]	A single institution experience of 141 cases of laparoscopic radical nephrectomy with cost-reductive measures	Article	Department of Urology, All India Institute of Medical Sciences, New Delhi, India	No
Aderounmu A.O.A.; et al. [20]	Rotational rural surgery for the poor in developing countries	Article	Department of Surgery, Lautech Teaching Hospital, Osogbo PMB 4400, Nigeria	Yes

**Table 2:** Number of citations from top 5 studies over the years.

Year	Document Title	Journal Title	Citations						
			<2017	2017	2018	2019	2020	2021	Total
			15	8	10	13	11	1	43
2011	Mesh fixation compared to nonfixation in total extraperitoneal inguinal hernia repair: A randomized controlled trial in a rural center in India	Surgical Endoscopy	10	3	4	5	3	0	15
2016	Acute postoperative pain in 1231 patients at a developing country referral hospital: Incidence and risk factors	Southern African Journal of Anaesthesia and Analgesia	0	1	1	4	5	0	11
2016	Outpatient laparoscopic nerve-sparing radical hysterectomy: A feasibility study and analysis of perioperative outcomes	Gynecologic Oncology	0	3	2	2	2	1	10
2009	A single institution experience of 141 cases of laparoscopic radical nephrectomy with cost-reductive measures	Journal of Endourology	5	0	3	1	0	0	4
2017	Single-incision laparoscopic surgery in gynecologic surgery: A single-institutional experience from Saudi Arabia	F1000Research	0	1	0	1	1	0	3
